# Psychological Flexibility With Prejudices Increases Empathy and Decreases Distress Among Adolescents: A Spanish Validation of the Acceptance and Action Questionnaire–Stigma

**DOI:** 10.3389/fpsyg.2020.565638

**Published:** 2021-01-22

**Authors:** Sonsoles Valdivia-Salas, José Martín-Albo, Araceli Cruz, Víctor J. Villanueva-Blasco, Teresa I. Jiménez

**Affiliations:** ^1^Department of Psychology and Sociology, University of Zaragoza, Teruel, Spain; ^2^Department of Psychology, Valencian International University, Valencia, Spain

**Keywords:** psychological inflexibility, psychological flexibility, stigma, empathic concern, personal distress, Spanish adolescents

## Abstract

Empathy is an emotional response that may facilitate prosocial behavior and inhibit aggression by increasing empathic concern for others. But the vicarious experience of other’s feelings may also turn into personal distress when the person has poor regulation skills and holds stigmatizing beliefs. In thinking about the processes that may trigger the experience of personal distress or empathic concern, research on the influence of psychological flexibility and inflexibility on stigma is showing promising results. Both processes are assessed with the Acceptance and Action Questionnaire–Stigma (AAQ-S). The current study sought to carry out a validity study of a Spanish version of the AAQ-S with a sample of adolescents aged 11–17 years. The study included an expanded test of its predictive validity with measures at three times to evaluate the role of psychological flexibility and inflexibility as risk or protective variables for the development of personal distress and/or empathic concern in the stigmatizer. Statistical analyses confirmed a two-correlated-factor solution, the adequate reliability of both factors, and their construct and predictive validity in the expected direction. The stigmatizer’s inflexible reaction to their stigmatizing thoughts predicted the occurrence of personal distress, whereas the stigmatizer’s flexible reaction to their stigmatizing thoughts predicted the occurrence of empathic concern for others. These findings confirm the importance of considering the role of regulatory skills in the experience of empathic concern or personal distress in the presence of stigmatizing thoughts, with possible implications for the promotion of prosocial behavior and the reduction of aggressive behavior among adolescents.

## Introduction

It is generally assumed that empathy is an emotional response that may facilitate prosocial behavior and inhibit aggression, thus leading to a better personal and social adjustment from childhood (e.g., Stavrinides et al., 2010; Wang et al., 2011; Mikolajewski et al., 2014; Carlo et al., 2015; Telle and Pfister, 2016; Deschamps et al., 2018; Tampke et al., 2020). The lack of consensus on the benefits of empathy (e.g., Vachon et al., 2014), however, has promoted the emergence of numerous models and theories about what empathy is and its relation with other constructs and outcomes (for a review, see Cuff et al., 2014).

The multidimensional model of empathy by Davis (1983), for instance, distinguishes between cognitive and affective empathy. Cognitive empathy is the ability to understand how others feel, whereas affective empathy entails the vicarious experience of others’ feelings (Vachon and Lynam, 2016). As noted by Eisenberg et al. (2010), if above some minimal threshold, such vicarious experience of other’s feelings may turn into empathic concern (EmpCon), personal distress (PerD), or both (see also Habashi et al., 2016). While EmpCon consists of feelings of sorrow or concern for the other, PerD is a self-focused, aversive–affective reaction to another’s emotion associated with the desire to alleviate one’s own, but not the other’s distress. Accordingly, EmpCon tends to be positively related to helping others, whereas PerD tends to be negatively or unrelated to prosocial behaviors (e.g., Graziano and Habashi, 2010; Habashi et al., 2016; Winter et al., 2017), also in adolescents (Eisenberg et al., 2010), and in Spanish adolescents (Gutiérrez et al., 2011; Tur-Porcar et al., 2016; Valdivia-Salas et al., 2021).

In thinking about the processes by which the vicarious experience of other’s feelings may evolve into either EmpCon or PerD, the mental flexibility and self-regulation component of empathy (Decety and Jackson, 2004) may shed some light. This component allows inhibiting one’s own perspective and engaging in perspective taking so as to evaluate the others’ point of view. These regulation skills prevent vicarious emotional hyperarousal from turning into PerD (Eisenberg, 2000; see also Valdivia-Salas et al., 2009; Ikezawa et al., 2012; Habashi et al., 2016; Grynberg and López-Pérez, 2018).

The PerD experienced within interpersonal domains may also result from holding stigmatizing beliefs about others (Masuda et al., 2009). Stigma is the tendency to evaluate and discriminate against others based on their group membership (Levin et al., 2014). Stigmatizing thoughts are usually regarded as rigid and self-protective because they facilitate the avoidance of perceived danger (Masuda et al., 2007; see also Kokkinos and Kipritsi, 2018). While there is extensive evidence of the harmful consequences of stigma for the stigmatized group (for a review, see Krafft et al., 2018), research conducted on the causes and consequences of stigma for the stigmatizer is rather scarce (Masuda et al., 2009). One line of such research suggests that psychological inflexibility (PsyInflex) may pose a risk for the development of PerD in the stigmatizer, which, in turn, may lead to the stigmatization of others (Masuda et al., 2009).

PsyInflex occurs when actions are rigidly guided by psychological reactions (e.g., perceived danger, negative affect, stigmatizing thoughts), rather than chosen values, and are primarily aimed at down-regulating such psychological reactions (e.g., Hayes et al., 1996; Törneke et al., 2008; Valdivia-Salas et al., 2010). PsyInflex is associated with a broad range of psychological and behavioral health problems, PerD among others (Monestès et al., 2018). On the contrary, its counterpart psychological flexibility (PsyFlex) refers to the process of engaging with psychological reactions without trying to down-regulate them, while acting in valued directions, and has been related to health and quality of life and life satisfaction (Monestès et al., 2018).

The empirical evidence on the relation between PsyInflex and stigma is scarce but shows consistent results. The development and validation of the questionnaire to assess PsyInflex and PsyFlex with prejudice thoughts, i.e., Acceptance and Action Questionnaire–Stigma (AAQ-S; Levin et al., 2014), were conducted with undergraduate students in a cross-sectional study and showed convergent validity with ethnocultural empathy, EmpCon, and perspective taking. A latter study revealed that the combination of high PsyInflex, low PsyFlex, low EmpCon, and low perspective taking accounted for approximately 36% of the variance in generalized prejudice and that the combination of PsyInflex and PsyFlex alone predicted generalized prejudice over right wing authoritarianism and social dominance (Levin et al., 2016). A recent review and meta-analysis (Krafft et al., 2018) has revealed significant correlations between PsyInflex and different types of stigma, with an overall effect size statistically significant and medium to large (see also Masuda et al., 2009).

All studies on the relation between PsyInflex and stigma have been conducted with North American adults, and their cross-sectional nature does not allow concluding about the mechanisms explaining this relation or the direction of the relations observed. In Spain, stigma has been explored primarily toward the mentally ill (e.g., Cangas et al., 2017), and only recently, there is a growing interest in exploring the potential of PsyInflex and PsyFlex with prejudice thoughts for the understanding of youngsters’ stigma toward the mentally ill (Trigueros et al., 2020). There is also evidence that general PsyInflex influences negatively Spanish children and adolescents’ psychological well-being (Valdivia-Salas et al., 2017; García-Gómez et al., 2019; Mestre et al., 2019; García-Rubio et al., 2020). But the relation among PsyFlex, PsyInflex, PerD, and EmpCon remains unexplored. In this direction, the current study sought to carry out a validity study of a Spanish version of the AAQ-S with a sample of adolescents aged 11–17 years. The study includes an expanded test of its predictive validity with measures at three times to evaluate the role of PsyInflex and PsyFlex as risk or protective variables for the development of PerD and EmpCon in the stigmatizer.

## Methods

### Participants

We employed randomized cluster sampling to select participants. The unit (cluster) was the school. The sampling frame was all the public schools in the target region. Each school on the list was assigned a weight equivalent to the number of students attending the school. Four schools were selected, and the principals of all of them agreed to participate. Students with ages ranging between 11 and 17 years (mean = 13.66, *SD* = 1.34) and attending the four compulsory secondary education courses filled out the questionnaires three times within a 12 months interval. The number of participants on each time was as follows: 847 (410 boys, 435 girls) at Time 1, 568 (271 boys, 297 girls) at Time 2, and 275 (131 boys, 144 girls) at Time 3.

### Measures and Instruments

#### PsyInflex and PsyFlex With Prejudice Thoughts

We employed an *ad hoc* Spanish translation of the AAQ-S (Levin et al., 2014). It includes 21 items that measure PsyFlex (“I am aware when judgments about others are passing through my mind,” “When I evaluate someone negatively, I am able to recognize that this is just a reaction, not an objective fact”) and PsyInflex (“My biases and prejudices affect how I interact with people from different backgrounds,” “When I have judgments about others, they are very intense”) with a broad range of negative thoughts about people belonging to different stigmatized groups. Response options range from 1 (*never true*) to 7 (*always true*), so that higher scores in the PsyInflex subscale indicate greater inflexibility, and higher scores in the PsyFlex subscale indicate greater flexibility. We translated the instrument into Spanish with the parallel back-translation procedure (Brislin, 1986). The items were first translated from English into Spanish by expert translators. The items were then back-translated into English and compared with the original ones. Finally, three experts and five students evaluated the adequacy of the items to the construct being assessed. We found no major difficulties with the semantic equivalence of the items in Spanish.

#### Psychological Inflexibility

We employed the Spanish validation (Valdivia-Salas et al., 2017) of the Avoidance and Fusion Questionnaire for Youth (AFQ-Y; Greco et al., 2008). The AFQ-Y contains 17 items, measuring two interrelated processes that characterize PI, namely, cognitive fusion (CF; e.g., “My thoughts and feelings mess up my life”) and experiential avoidance (EA; e.g., “I stop doing things that are important to me whenever I feel bad”), and respondents rate how true each statement is for them on a 5-point scale ranging from 0 (*not at all true*) to 4 (*very true*). Higher scores indicate greater CF and EA. The Spanish validation showed good psychometric properties with Spanish adolescents (Valdivia-Salas et al., 2017).

#### Empathy

We employed the Spanish validation (Pérez-Albéniz et al., 2003) of the Interpersonal Reactivity Index (IRI; Davis, 1980). The IRI is a 28-item self-report questionnaire that comprised four subscales, namely, Fantasy, Perspective Taking, EmpCon, and PerD (seven items each). We employed only the EmpCon and PerD subscales because of the literature that poses them as variables involved in either prosocial or aggressive behavior (Cuff et al., 2014). The EmpCon reflects the affective/emotional component of empathy and assesses the tendency to experience warmth, concern, and compassion for others (e.g., “I often have tender, concerned feelings for people less fortunate than me,” “I would describe myself as a pretty soft-hearted person”). The PerD subscale assesses feelings of discomfort and anxiety that people may experience when they witness negative experiences in others (e.g., “I feel helpless when I am in an emotionally charged situation, “It scares me to be in a tense emotional situation”). Responders rate each item on a scale ranging from 0 (*not at all like me*) to 4 (*very much like me*), and items are summed so that higher scores reflect greater EmpCon and greater PerD. There is evidence of the good psychometric properties of these two subscales in Spanish adolescents (Carrasco Ortiz et al., 2011).

### Procedure

We contacted the principals of the target schools to explain the purpose of the research and to request their permission to carry out the study. After we had obtained permission from the school principals, we requested the parents’/tutors’ consent for their children to participate in the study. Once in the classroom, the researchers described the goals of the study and informed the students that their participation was voluntary and confidential and that there were no good or bad answers. At least one researcher was present during the administration of the instruments to provide students with the necessary support to successfully complete the instruments.

### Data Analysis

We first calculated the descriptive statistics (mean, *SD*, and correlations) of the AAQ-S items and their mean differences as a function of gender by using the statistical package SPSS for Windows version 22 (IBM Corp, 2013).

The factorial structure of the scale was analyzed with the weighted least squares mean and variance adjusted estimation of 6.1 Mplus (Muthén and Muthén, (1998-2010)). The fact that classroom was our sample unit violated the assumption of independence of observations. This might have inflated the χ^2^ value and underestimated standard errors (Stapleton, 2006). For this reason, the “cluster” option for classrooms and COMPLEX function were used in all analyses. We used Cronbach α to test the reliability of the AAQ-S and the results of the confirmatory factor analysis (CFA) to calculate the composite reliability index (CRI).

**FIGURE 1:**
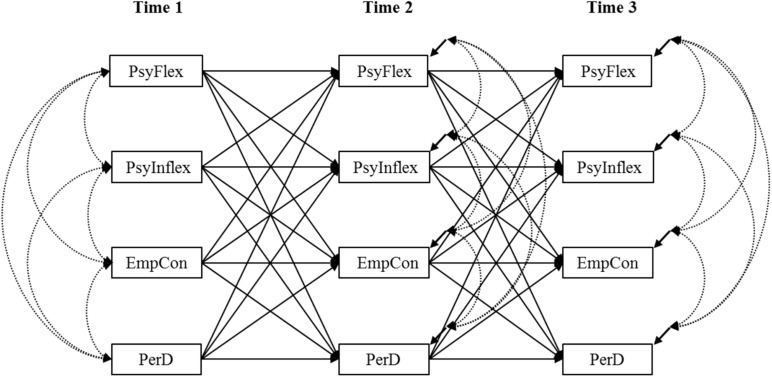
Longitudinal path.

In order to test for the construct validity of the AAQ-S, we conducted a new CFA model incorporating the two factors of the AFQ-Y. In order to establish the AAQ-S predictive validity, we tested the model shown in Figure 1 with longitudinal path analysis methodology and the maximum-likelihood robust estimation.

The following goodness-of-fit indices were reported: χ^2^ for model fit, root mean square error of approximation (RMSEA), comparative fit index (CFI), and Tucker–Lewis Index (TLI). Following Browne and Cudeck (1993) indications, RMSEA values close to 0.05, close to 0.08, and higher than 0.10 indicate good, reasonable, and bad fit, respectively. CFI values should be higher than 0.90 (Marsh et al., 2004). TLI values between 0.90 and 0.95 are considered acceptable. Finally, missing data were replaced by the method of linear interpolation usually employed by Mplus.

## Results

### Descriptive Analyses

As Table 1 shows, mean PsyInflex scores (items 1, 4, 6, 7, 8, 9, 13, 15, 16, 20, 21) were, overall, lower than mean PsyFlex scores (items 2, 3, 5, 10, 11, 12, 14, 17, 18, 19). All PsyInflex items scored lower than 2.5, whereas all PsyFlex items, except for items 12 and 18, scored higher than 2.5. As for the correlations, they were strong among same factor items, with some exceptions: item 4 correlated positively with item 5 (*r*_xy_ = 0.37; *p* < 0.05) and item 19 (*r*_xy_ = 0.27; *p* < 0.05); as well, item 16 correlated positively with item 17 (*r*_xy_ = 0.32; *p* < 0.05). Kurtosis and asymmetry indices were lower than 2, which prove univariate normality.

**TABLE 1:** Descriptive analyses, correlations, variances, and covariances.

Items*	1	2	3	4	5	6	7	8	9	10	11	12	13	14	15	16	17	18	19	20	21
1.	1.07	0.23**	0.19**	0.19**	0.10**	0.31**	0.33**	0.27**	0.30**	0.20**	0.17**	0.23**	0.22**	0.16**	0.23**	0.19**	0.09**	0.18**	0.24**	0.25**	0.15**
2.	0.31	1.61	0.29**	0.26**	0.30**	0.10**	0.21**	0.14**	0.19**	0.40**	0.26**	0.15**	0.12**	0.27**	0.13**	0.27**	0.36**	0.11**	0.29**	0.18**	0.15**
3.	0.27	0.50	1.92	0.15**	0.30**	0.08**	0.11**	0.04**	0.10**	0.34**	0.26**	0.14**	0.05	0.23**	0.16**	0.16**	0.30**	0.08*	0.29**	0.10**	0.05
4.	0.27	0.46	0.30	1.97	0.37**	0.15**	0.33**	0.13**	0.30**	0.22**	0.20**	0.13**	0.09*	0.16**	0.16**	0.14**	0.21**	0.08**	0.27**	0.24**	0.14**
5.	0.15	0.53	0.58	0.72	1.98	0.01	0.18**	−0.03**	0.09**	0.38**	0.27**	0.08*	–0.00	0.29**	0.08*	0.19**	0.36**	0.04	0.27**	0.17**	0.03
6.	0.33	0.13	0.11	0.21	0.01	1.08	0.30**	0.30**	0.32**	0.10**	0.08*	0.20**	0.37**	0.17**	0.26**	0.11**	0.02	0.25**	0.09**	0.17**	0.19**
7.	0.37	0.30	0.17	0.50	0.27	0.34	1.20	0.23**	0.44**	0.20**	0.19**	0.22**	0.26**	0.18**	0.27**	0.15**	0.15**	0.21**	0.23**	0.25**	0.15**
8.	0.29	0.18	0.05	0.18	–0.04	0.32	0.26	1.04	0.38**	0.11**	0.07	0.21**	0.21**	0.07*	0.30**	0.16**	0.02	0.26**	0.12**	0.17**	0.19**
9.	0.35	0.28	0.16	0.47	0.15	0.38	0.55	0.44	1.28	0.18**	0.14**	0.22**	0.27**	0.20**	0.30**	0.21**	0.12**	0.29**	0.29**	0.17**	0.20**
10.	0.28	0.68	0.63	0.42	0.72	0.13	0.30	0.16	0.27	1.81	0.33**	0.18**	0.06	0.38**	0.19**	0.29**	0.52**	0.15**	0.40**	0.24**	0.17**
11.	0.24	0.46	0.50	0.38	0.53	0.11	0.29	0.09	0.22	0.62	1.91	0.19**	0.07*	0.25**	0.20**	0.18**	0.31**	0.08*	0.38**	0.24**	0.16**
12.	0.29	0.24	0.24	0.22	0.15	0.25	0.30	0.27	0.31	0.30	0.33	1.54	0.24**	0.21**	0.27**	0.15**	0.12**	0.29**	0.27**	0.21**	0.14**
13.	0.22	0.14	0.07	0.12	–0.00	0.36	0.27	0.21	0.28	0.07	0.10	0.28	0.90	0.19**	0.28**	0.14**	0.05	0.23**	0.08*	0.17**	0.17**
14.	0.23	0.46	0.43	0.30	0.55	0.24	0.26	0.10	0.30	0.69	0.47	0.36	0.24	1.80	0.30**	0.29**	0.37**	0.16**	0.34**	0.19**	0.18**
15.	0.26	0.18	0.25	0.25	0.12	0.30	0.33	0.34	0.38	0.28	0.31	0.37	0.29	0.45	1.23	0.23**	0.22**	0.29**	0.23**	0.24**	0.29**
16.	0.25	0.44	0.29	0.27	0.36	0.14	0.22	0.22	0.31	0.51	0.32	0.24	0.17	0.50	0.33	1.72	0.32**	0.23**	0.29**	0.27**	0.20**
17.	0.13	0.61	0.55	0.39	0.67	0.03	0.21	0.03	0.18	0.93	0.58	0.20	0.06	0.66	0.33	0.56	1.78	0.16**	0.46**	0.24**	0.18**
18.	0.22	0.17	0.14	0.13	0.07	0.30	0.27	0.32	0.38	0.24	0.14	0.43	0.26	0.26	0.39	0.36	0.25	1.43	0.22**	0.21**	0.23**
19.	0.33	0.50	0.54	0.53	0.53	0.13	0.35	0.17	0.45	0.74	0.72	0.45	0.10	0.62	0.36	0.53	0.83	0.35	1.87	0.29**	0.25**
20.	0.32	29	0.17	0.42	0.29	0.22	0.33	0.22	0.24	0.40	0.42	0.33	0.20	0.32	0.34	0.44	0.40	0.31	0.50	1.54	0.31**
21.	0.17	0.21	0.07	0.21	0.05	0.21	0.18	0.21	0.24	0.25	0.25	0.20	0.18	0.28	0.35	0.29	0.28	0.30	0.38	0.42	1.23
Mean:	1.91	3.06	2.57	2.48	2.85	1.76	1.96	1.64	1.92	2.93	2.72	2.20	1.62	5.56	1.93	2.47	2.80	2.03	2.80	2.25	2.04
*SD*:	1.03	1.27	1.38	1.40	1.41	1.04	1.10	1.02	1.13	1.34	1.38	1.24	0.95	1.34	1.11	1.31	1.33	1.20	1.37	1.24	1.11
Skewness:	1.13	−0.06	0.43	0.49	0.14	1.37	1.06	1.69	1.15	0.05	0.31	0.77	1.65	0.45	1.15	0.56	0.18	1.00	0.21	0.75	0.95
Kurtosis:	0.84	−0.99	–1.09	–1.07	–1.26	1.21	0.48	2.27	0.49	–1.15	–1.13	–0.46	2.34	–0.95	0.61	–0.78	–1.12	0.04	–1.16	–0.44	0.23

SD, Standard Deviation. *p < 0.05. **p < 0.01.^∗^Items (Spanish adaptation)

*1. My biases and prejudices affect how I interact with people from different backgrounds (Mis prejuicios influyen en mi manera de relacionarme con personas que son diferentes a mí).*

*2. I feel that I am aware of my own biases (Siento que soy consciente de mis propios prejuicios).*

*3. My negative thoughts about others are never a problem in my life (Mis pensamientos negativos sobre otras personas no me generan problemas en mi vida).*

*4. I need to reduce my negative thoughts about others in order to have good social interactions (Necesito reducir los pensamientos negativos hacia los demás para poder tener buenas relaciones con ellos).*

*5. When I evaluate someone negatively, I am able to recognize that this is just a reaction, not an objective fact (Cuando pienso mal sobre alguien, soy capaz de reconocer que esto es solo lo que yo pienso, y no necesariamente algo real sobre esa persona).*

*6. I stop doing things that are important to me when it involves someone I don’t like (Dejo de hacer las cosas que son importantes para mí cuando participa alguien que no me gusta).*

*7. I have trouble letting go of my judgments of others (Me resulta difícil conseguir que mis juicios sobre los demás no me influyan).*

*8. I feel that my prejudicial thoughts are a significant barrier to me being culturally sensitive (Siento que mis prejuicios me dificultan respetar a personas de otras culturas).*

*9. I have trouble not acting on my negative thoughts about others (Me resulta complicado que mis pensamientos negativos sobre los demás no me influyan en cómo actúo).*

*10. I’m good at noticing when I have a judgment of another person (Cuando hago juicios sobre otras personas, me doy cuenta de que los estoy haciendo).*

*11. It’s OK to have friends that I have negative thoughts about from time to time (No pasa nada por tener amigos sobre los que a veces tengo pensamientos negativos).*

*12. I don’t struggle with controlling my evaluations about others (No me esfuerzo por evitar hacer valoraciones sobre los demás).*

*13. When I am having negative thoughts about others, I withdraw from people (Cuando tengo pensamientos negativos sobre otras personas, me aislo de la gente).*

*14. When I’m talking with someone I don’t like, I’m aware of my evaluations of them (Cuando estoy hablando con alguien que no me gusta, me doy cuenta de las valoraciones negativas que hago de esa persona).*

*15. When I have judgments about others, they are very intense (Cuando juzgo a los demás, suelo hacerlo de manera muy intensa).*

*16. When talking with someone I believe should act according to how I feel about him/her, even if it is negative (Creo que cuando hablo con una persona, yo debería actuar de acuerdo a lo que siento por ella, incluso cuando lo que siento es negativo).*

*17. I am aware when judgments about others are passing through my mind (Se me da bien darme cuenta cuando hago juicios sobre otras personas).*

*18. I rarely worry about getting my evaluations toward others under control (No me preocupo por mantener bajo control mis valoraciones negativas sobre los demás).*

*19. I accept that I will sometimes have unpleasant thoughts about other people (Acepto que a veces tendré pensamientos desagradables sobre los demás).*

*20. I often get caught up in my evaluations of what others are doing wrong (A menudo me quedo pillado dándole vueltas a las valoraciones que hago sobre lo que otros hacen mal).*

*21. The bad things I think about others must be true (Deben de ser verdad las cosas malas que pienso de los demás).*

### Confirmatory Factor Analysis and Reliability

Following the original structure, we tested the two-correlated-factor solution, which showed good fit indices in the total sample [χ^2^ = 500.36, degrees of freedom (DF) = 188, RMSEA (low, high) = 0.04 (0.04, 0.05), CFI = 0.91, TLI = 0.91] and in both boys [χ^2^ = 514.36, DF = 188, RMSEA (low, high) = 0.06 (0.06, 0.07), CFI = 0.92, TLI = 0.91] and girls [χ^2^ = 355.69, DF = 188, RMSEA (low, high) = 0.05 (0.04, 0.05), CFI = 0.92, TLI = 0.91]. As shown in Table 2, all standardized parameters were positive and significant (*p* < 0.05) and greater than 0.40 except for item 6 (β = 0.39).

**TABLE 2:** Estimated parameters in the confirmatory factor analysis.

	Item	Estimate	*SE*	Standardized estimate	Standardized *SE*
Inflex by	T1PRE_1	1.000	0.000	0.605	0.014
	T1PRE_4	0.876	0.056	0.530	0.026
	T1PRE_6	0.638	0.028	0.386	0.021
	T1PRE_7	1.101	0.060	0.666	0.028
	T1PRE_8	0.795	0.090	0.481	0.046
	T1PRE_9	1.085	0.059	0.657	0.021
	T1PRE_13	0.680	0.020	0.412	0.009
	T1PRE_15	1.090	0.054	0.660	0.020
	T1PRE_16	1.091	0.063	0.660	0.032
	T1PRE_20	0.926	0.032	0.560	0.011
	T1PRE_21	0.905	0.068	0.548	0.030
Flex by	T1PRE_2	1.000	0.000	0.531	0.015
	T1PRE_3	0.900	0.079	0.478	0.030
	T1PRE_5	0.976	0.039	0.518	0.013
	T1PRE_10	1.393	0.064	0.739	0.028
	T1PRE_11	0.967	0.046	0.514	0.020
	T1PRE_12	0.947	0.026	0.503	0.006
	T1PRE_14	1.113	0.068	0.591	0.021
	T1PRE_17	1.300	0.059	0.690	0.021
	T1PRE_18	0.774	0.028	0.411	0.020
	T1PRE_19	1.342	0.044	0.713	0.018
Flex with inflex		0.224	0.007	0.697	0.008

*All parameters were significant with p < 0.01.*

Reliability, as measured with Cronbach α, was adequate for both PsyFlex (α = 0.80) and PsyInflex (α = 0.77). Considering that α values may underestimate the real population value, we calculated the CRI (Graham, 2006), which resulted in reliability values of 0.83 for both PsyFlex and PsyInflex.

### Construct Validity

Cronbach α for the two factors of the AFQ-Y was 0.82 for CF and 0.78 for EA. Results of the four correlated factor model were good [χ^2^ = 1,001.93, DF = 622, RMSEA (low, high) = 0.03 (0.02,0.03), CFI = 0.92, TLI = 0.92]. As expected, the two factors of the AFQ-Y showed more strong and positive relations with PsyInflex (*r*_xy_ = 0.64 for CF; and *r*_xy_ = 0.57 for EA) than with PsyFlex (*r*_xy_ = 0.30 for CF; and *r*_xy_ = 0.24 for EA).

### Predictive Validity

We first conducted CFA and reliability calculations for PerD and EmpCon subscales. The model showed adequate fit indices [χ^2^ = 342.22, DF = 76, RMSEA (low, high) = 0.06 (0.05, 0.07), CFI = 0.94, TLI = 0.93]. Cronbach α was 0.70 for EmpCon and 0.67 for PerD, and CRI was 0.86 for EmpCon and 0.78 for PerD. The path model showed good fit indices [χ^2^ = 55.71, DF = 16, RMSEA (low, high) = 0.05 (0.04, 0.07), CFI = 0.97, TLI = 0.91].

#### Direct Effects

As shown in Table 3, all autoregressive parameters were positive and significant, which showed the temporal stability of the four factors. Regarding the predictive relation between PsyFlex, PsyInflex, and both PerD and EmpCon, the results showed that, from Time 3 to Time 2, PerD was positively related to PsyInflex (β = 0.15, *p* < 0.05), and EmpCon was positively related to PsyFlex (β = 0.11, *p* < 0.05) and negatively related to PsyInflex (β = −0.09, *p* < 0.05). Similar patterns of relations occurred from Time 2 to Time 1 (Table 3). Interestingly, while the relation between PerD and PsyInflex was unidirectional, with PsyInflex predicting PerD, the relation between EmpCon and PsyFlex was bidirectional (Table 3). The explained variance of the model was 34% for EmpCon, 36% for PerD, 30% for PsyFlex, and 26% for PsyInflex.

**TABLE 3:** Estimated parameters in the longitudinal path model.

	Variables	Estimate	*SE*	Standardized estimates	*SE*	*p*
PerDT3 ON	PerDT2	0.513	0.047	0.551	0.076	0.000
	EmpConT2	–0.059	0.043	–0.063	0.051	0.213
	PsyFlexT2	0.041	0.030	0.047	0.038	0.225
	PsyInflexT2	0.156	0.045	0.147	0.033	0.000
EmpConT3 ON	PerDT2	0.179	0.083	0.171	0.092	0.064
	EmpConT2	0.456	0.056	0.433	0.006	0.000
	PsyFlexT2	0.110	0.001	0.112	0.012	0.000
	PsyInflexT2	–0.105	0.004	–0.088	0.011	0.000
PsyFlexT3 ON	PerDT2	–0.066	0.052	–0.062	0.048	0.194
	EmpConT2	0.222	0.085	0.206	0.089	0.020
	PsyFlexT2	0.494	0.036	0.493	0.013	0.000
	PsyInflexT2	–0.097	0.099	–0.079	0.084	0.342
PsyInflexT3 ON	PerDT2	0.011	0.066	0.014	0.080	0.865
	EmpConT2	0.036	0.027	0.043	0.037	0.247
	PsyFlexT2	–0.007	0.017	–0.008	0.023	0.713
	PsyInflexT2	0.479	0.111	0.501	0.064	0.000
PerDT2 ON	PerDT1	0.490	0.051	0.470	0.048	0.000
	EmpConT1	0.093	0.040	0.091	0.038	0.015
	PsyFlexT1	0.023	0.045	0.024	0.047	0.603
	PsyInflexT1	0.083	0.039	0.069	0.036	0.053
EmpConT2 ON	PerDT1	0.048	0.045	0.046	0.044	0.294
	EmpConT1	0.478	0.018	0.472	0.015	0.000
	PsyFlexT1	0.134	0.023	0.141	0.020	0.000
	PsyInflexT1	–0.037	0.078	–0.031	0.065	0.633
PsyFlexT2 ON	PerDT1	0.033	0.035	0.030	0.031	0.338
	EmpConT1	0.092	0.009	0.085	0.008	0.000
	PsyFlexT1	0.373	0.020	0.366	0.013	0.000
	PsyInflexT1	0.038	0.072	0.030	0.057	0.603
PsyInflexT2 ON	PerDT1	0.068	0.030	0.074	0.033	0.025
	EmpConT1	0.028	0.041	0.031	0.046	0.491
	PsyFlexT1	–0.131	0.008	–0.156	0.012	0.000
	PsyInflexT1	0.503	0.016	0.478	0.014	0.000

*PerD, personal distress; EmpCon, emphatic concern; PsyFlex, psychological flexibility; PsyInflex, psychological inflexibility; T1, Time 1; T2, Time 2; T3, Time 3.*

#### Indirect Effects

The analyses confirmed two routes leading to PerD in Time 3: on the one hand, the PsyInflex route, starting at Time 1 and going through Time 2, which yielded a positive relation [β = 0.08, *SE* = 0.03 (standardized = 0.07; 0.02); *p* < 0.01]; on the other hand, the mixed route, starting with PsyFlex at Time 1 and going through PsyInflex at Time 2, which yielded a negative relation for the occurrence of PerD [β = −0.02; *SE* = 0.006; (standardized = −0.02; 0.006) *p* < 0.01]. In both cases, PsyInflex played a significant mediating role. Regarding the occurrence of EmpCon at Time 3, analyses confirmed three routes: first, the PsyInflex route, starting at Time 1 and going through Time 2, which yielded a negative relation [β = −0.05; *SE* = 0.001; (standardized = −0.04; 0.004); *p* < 0.01]; the second route started with PsyFlex at Time 1 and continued with EmpCon at Time 2, with positive effect on EmpCon at Time 3 [β = 0.06; *SE* = 0.004; (standardized = 0.06;0.008); *p* < 0.01]; lastly, the PsyFlex route, which also had a positive effect on EmpCon at Time 3 and had PsyFlex as predictive variable at both Times 1 and 2 [β = 0.04; *SE* = 0.004; (standardized = −0.04; 0.01); *p* < 0.01]. These EmpCon routes indicate the mediating role of PsyFlex, although in this case, PsyFlex and EmpCon seem to show a non-recursive pattern of influence.

## Discussion

The purpose of the current research was to conduct a validity study of a Spanish version of the AAQ-S with a sample of adolescents aged 11–17 years. Statistical analyses confirmed a two-correlated-factor solution (PsyFlex and PsyInflex), the adequate reliability of both factors and their construct and predictive validity in the expected direction.

The CRI yielded the same reliability for both PsyFlex and PsyInflex, a value similar to that shown in the initial validation of the instrument (Levin et al., 2014). Still, the PsyInflex subscale showed only adequate α value, and the percentage of explained variance of the predictive model was lower than that of PsyFlex. This may be the result of the wording of the PsyInflex items that may have been difficult for early adolescents (in our sample, average age was 13.66 years; age range, 11–17 years). In fact, in the study by Trigueros et al. (2020), which employed an older sample (average age was 17.12 years; age range, 15–19 years), reliability of the PsyInflex subscale was higher. Should this instrument be further adapted to early adolescence, rewording of the PsyInflex items is highly recommended (Greco et al., 2008).

Most items loaded onto their corresponding factor, but we also found unexpected correlations among different-factor items. Again, we argue that the adaptation of this instrument to early adolescents require further refinement in the wording of the items so as to clearly differentiate between flexible and inflexible reactions to stigmatizing thoughts. For instance, PsyFlex item 17 reads: “I am good at noticing when I have a judgment of another person,” and PsyInflex item 16 reads: “When talking with someone, I believe I should act according to how I feel about him/her, even if it is negative.” However, it is compatible being aware of your stigmatizing thoughts and still acting on them. Unfortunately, in the original validation of the scale (Levin et al., 2014), PsyFlex items were reverse scored, so it is not possible to establish comparisons with our findings. Further refinements of the instrument adapted to Spanish early adolescents ought to take this into consideration.

The two-factor solution showed good fit indices in the total sample and when separated by gender. This goes in line with previous studies that have not found differences in PsyInflex and PsyFlex with stigmatizing thoughts as a function of gender (Trigueros et al., 2020). Regardless of how the stigmatizing thoughts are acquired and how their content may be different as a matter of gender, our results suggest that both genders relate to such thoughts both in flexible and inflexible ways. This probably relates to the fact that PsyInflex and PsyFlex are general overarching regulatory skills that, once learned, occur with thoughts and feelings that are experienced as aversive regardless of their content (Monestès et al., 2018). The construct validity study showed that the inflexibility factors of the AFQ-Y, namely, CF and EA, were more strongly related to PsyInflex than to PsyFlex, as expected.

The predictive validity study yielded the most interesting results. As expected, direct effects showed that PsyInflex predicted the occurrence of PerD and prevented the occurrence of EmpCon, and PsyFlex predicted the occurrence of EmpCon. PsyInflex with stigmatizing thoughts leads to PerD and blocks the experience of empathy in the stigmatizer. Interestingly, while the relation between PsyInflex and PerD seems unidirectional, with PsyInflex predicting PerD, the relation between PsyFlex and EmpCon seems bidirectional. That is, relating with flexibility to our stigmatizing thoughts about others makes us more empathetic, and in turn, practicing the empathetic concern for others’ situation makes us more empathetic and more psychologically flexible with our stigmatizing thoughts, in a kind of upward virtuous cycle. The bidirectional relation between psychological in/flexibility and outcome variables has been reported before (Valdivia-Salas et al., 2017) and shows its susceptibility to training and change over time. This is especially relevant during adolescence, when regulatory skills are not yet fully consolidated, and hence prevention and socioeducative interventions are crucial (Glick and Hilt, 2000).

In the same line, the indirect effects showed expected results such as the predictive role of PsyInflex for the development of PerD and the predictive role of PsyFlex for the development of EmpCon. This suggests that, when maintained over time as the predominant coping strategy, PsyInflex will lead to PerD, whereas PsyFlex will lead to EmpCon. Indirect effect also showed the protective effects of PsyFlex for the development of PerD in that high PsyInflex scores at Time 2 led to either PerD or EmpCon, depending on the behavior regulation skills at Time 1: when adolescents scored high in PsyFlex at Time 1, the most probable outcome at Time 3 was EmpCon, whereas when adolescents scored high in PsyInflex at Time 1, the most probable outcome at Time 3 was PerD. Somehow, departing from flexible regulatory skills buffered the negative consequences of developing inflexible regulatory skills at some time during development. This has great implications for the promotion of PsyFlex as early as possible during childhood. And calls for further research on the role exerted by PsyFlex in the interplay among PerD, EmpCon, and perspective-taking, one of the components of cognitive empathy, which seems to deactivate PerD in favor of EmpCon (Graziano and Habashi, 2010; Habashi et al., 2016). It might be the case that PsyFlex, which involves perspective from our own thoughts (Levin et al., 2014), somehow facilitated taking another’s perspective and led to EmpCon rather than PerD.

These findings confirm the importance of considering the role of regulatory skills in the experience of either EmpCon or PerD when in the presence of stigmatizing thoughts about others (Decety and Jackson, 2004). And also, these findings go in line with the evidence that acting on thoughts and feelings to somehow alter their content or alleviate their presence (i.e., PsyInflex) leads to internalizing and externalizing problems, also during adolescence. As reviewed in the introductory section, PerD is regarded as a self-focus aversive–affective reaction to another’s emotion that is associated with the desire to alleviate one’s own, but not the other’s distress (e.g., Eisenberg et al., 2010; Winter et al., 2017). Our findings prove that PerD derives from identifying with stigmatizing thoughts and acting on them (or “pushed” by them) despite the mid- and long-term consequences this may have in both the stigmatizer and the stigmatized.

Our study demonstrates that behavior regulation skills influence the degree to which an adolescent will experience more EmpCon or more PerD when they witness a negative experience of others. But we cannot assert that PsyFlex and PsyInflex will influence their engagement in either prosocial or aggressive behavior. Previous literature would support this hypothesis (see, for instance, Masuda et al., 2007, 2009); still further research is called for. Further research should also include larger samples so as to increase the generalizability of our findings, additional waves that allowed analyzing within-subject and between-subject effects, and experimental designs.

In Spain, prejudices and stigma become evident in the behavior of adolescents from the early age of 12 years (e.g., Ferragut et al., 2017). Our study shows that the way the adolescents react to their stigmatizing thoughts may be one early link to the chain of either prosocial or aggressive/discriminatory behavior.

## Data Availability Statement

The raw data supporting the conclusions of this article will be made available by the authors, without undue reservation.

## Ethics Statement

The studies involving human participants were reviewed and approved by Comité ético de investigación clínica de Aragón. Written informed consent to participate in this study was provided by the participants’ legal guardian/next of kin.

## Author Contributions

SV-S and JM-A designed the study. VV-B managed data collection and database update. AC assisted during data collection. JM-A conducted data analyses. SV-S, JM-A, and AC wrote the manuscript. VV-B and TJ revised previous versions of the manuscript. All authors read and approved the submitted version.

## Conflict of Interest

The authors declare that the research was conducted in the absence of any commercial or financial relationships that could be construed as a potential conflict of interest.
